# DESIGN AND IMPLEMENTATION OF THE ACHIEVE HEARING INTERVENTION

**DOI:** 10.1093/geroni/igad104.0297

**Published:** 2023-12-21

**Authors:** Theresa Chisolm, Victora Sanchez, Michelle Arnold

**Affiliations:** University of South Florida, Tampa, Florida, United States; University of South Florida, Tampa, Florida, United States; University of South Florida, Tampa, Florida, United States

## Abstract

The ACHIEVE trial required the development of a manualized best-practice hearing intervention designed to meet the needs of older adults with mild-moderate hearing loss. The hearing intervention (HI) was developed using an iterative process including input from a diverse professional panel and a feasibility study to confirm that expected outcomes from a best-practices, evidence-based HI would be obtained. The HI involves use of hearing aids and related technologies along with counselling, coaching and education to support self-management of hearing loss. The HI is delivered in four sessions after the completion of a comprehensive hearing evaluation. Individualized goals are developed using the Client Oriented Scale of Improvement (COSI), which guides selection of hearing aids and other technologies to meet prioritized listening needs and assessment of how well those needs are met. The most frequent COSI listening goal categories included: Conversation in Noise (n= 413; 29%), Conversations in Quiet (n= 296; 21%), and TV/Radio at Normal Volume (n= 280; 20%). Three levels of hearing aid technology differing by the number of signal processing approaches included were: Basic (n=223, 20%), Advanced (n=662, 56%), and Premium (n=283, 24%). Additional hearing assistive technologies included streaming devices, TV connectors, and remote microphones were provided. We discuss how the baseline audiological results and individualized goal setting informed delivery of the HI and how the use of manualization allowed us to maintain excellent treatment fidelity throughout the clinical trial.

